# Knowledge and Awareness of Performance Metrics in Dental Image Analysis With Artificial Intelligence: A Cross-Sectional Survey of Researchers and Clinicians

**DOI:** 10.1016/j.identj.2026.109566

**Published:** 2026-05-06

**Authors:** Rishi Ramani, Bree Jones, Akhilanand Chaurasia, Yeganeh Khazaei, Julia Schwärzler, Sergio E. Uribe, Falk Schwendicke, Antonin Tichy

**Affiliations:** a Dental Data and Artificial Intelligence (D2AI) research group, Melbourne Dental School, University of Melbourne, Victoria, Australia; b Topic Group Oral Health, ITU/WHO/WIPO Global Initiative on Artificial Intelligence for Health, Geneva, Switzerland; c Department of Oral Medicine and Radiology, King George’s Medical University, Lucknow, India; d Clinic for Conservative Dentistry, Periodontology and Digital Dentistry, LMU University Hospital, Munich, Germany; e Department of General Dentistry, Rīga Stradiņš University, Rīga, Latvia; f Institute of Stomatology, Rīga Stradiņš University, Rīga, Latvia; g Institute of Dental Medicine, First Faculty of Medicine, Charles University, Prague, Czech Republic

**Keywords:** Artificial intelligence, Radiography, dental, digital, Surveys and questionnaires, Sensitivity and specificity, Health knowledge, attitudes, practice, Predictive value of tests

## Abstract

**Introduction and aim:**

Appropriate performance metrics are essential for evaluating machine learning (ML) algorithm performance in dental image analysis. A broad variety of performance metrics are used in dental artificial intelligence (AI) research which could cause confusion regarding model performance. This study aimed to assess the knowledge, awareness, and self-reported confidence of oral health researchers and clinicians regarding AI performance metrics.

**Methods:**

This cross-sectional, questionnaire-based survey initially recruited participants (*n* = 100) from the ITU/WHO/WIPO Global Initiative on AI for Health Topic Group – Oral Health and affiliated professional global networks. An online self-administered questionnaire evaluated theoretical knowledge and applied knowledge. Participants also reported basic demographics and their confidence in using different metrics.

**Results:**

Overall performance was poor with participants achieving a mean score of 17.1% (SD: 15.2%, median: 16.7%, IQR: 16.7%) for fully correct responses. Responses to theoretical questions were rarely fully correct (range: 1%-15%; median: 5.5%). Applied scenario performance ranged from 17% to 37% correct responses (median: 29%). Participants demonstrated greater knowledge of common metrics (accuracy, specificity) but struggled with advanced measures (Intersection over Union & Dice Similarity Coefficient). Common errors included terminology confusion, familiarity bias towards accuracy, and task-metric misalignment.

**Conclusion:**

Substantial gaps exist in knowledge and awareness of both theory and practical applications of AI performance metrics amongst dental professionals and researchers. Frequent misapplication of commonly known metrics suggests overgeneralisation without consideration of task-specific requirements.

**Clinical Relevance:**

Inadequate knowledge or awareness of metric selection and interpretation may lead to the deployment of suboptimal algorithms, which can compromise diagnostic accuracy and patient outcomes. Enhanced understanding of performance metrics is critical for ensuring reliable, fair, safe, and effective AI systems in clinical dentistry.

## Introduction

Evaluating performance and progress in any field relies on the use of appropriate performance metrics. In healthcare, the concordance between selected performance metrics and the intended clinical outcome is critical, as metrics directly influence how algorithm performance is interpreted and translated into clinical decisions.^1^ Different image analysis tasks in healthcare require attention to distinct aspects of model performance, and inappropriate metric selection can obscure true suitability for clinical use, promote suboptimal solutions, and misdirect research and implementation efforts.^2^

These concerns are particularly relevant for machine learning (ML), which is a subfield of artificial intelligence (AI). In dental image analysis, AI and ML models are increasingly evaluated based on technical model development rather than from clinical decision-making frameworks.^3^^,^^4^ This pattern has been driven by advances in education, accessibility, affordability, computing power, and successes in other medical fields such as mammography.^5^ The leap from academic studies to clinical application risks creating a knowledge gap among clinicians and researchers about the theoretical underpinnings of ML. A recent review found that less than half of the ML papers in dentistry adequately explained their results in relation to model performance metrics, raising concerns about both research transparency and clinical translation.^6^ Technical performance measures are often implicitly treated as indicators of clinical impact, despite not capturing downstream clinical consequences.^4^ For example, a diagnostic model reporting very high sensitivity may be clinically uninformative in low-prevalence settings if specificity is poor, as this would generate large numbers of false positives and unnecessary follow-up procedures, limiting clinical usefulness despite apparently strong performance.^7^

Recent work in medical AI has highlighted widespread confusion in metric selection and interpretation, prompting the development of frameworks. Metric selection, once treated as a technical requirement, has moved to the centre of discussion in medical AI, driven by efforts to make its principles clearer and more consistent. Maier-Hein et al^8^ helped lead this change with the Metrics Reloaded framework, which guides researchers to choose performance metrics that genuinely reflect the biomedical question rather than relying on habit. Reinke et al^2^ exposed just how much confusion still surrounds common metrics and created a practical resource to help address it. Together, these studies show that misunderstandings about how to validate AI systems adequately persist in biomedical applications.

A range of metrics can be used to evaluate performance of ML tasks in dental imaging as reported by the recent dental computer vision core outcome measures (DentalCOMS) study.^9^ Using a small subset of performance metrics for specific tasks in medicine and dentistry risks giving a misleading impression of the model’s actual capabilities. This could lead to unexpected outcomes in a clinical setting and increase the risk of confirmation bias when reporting on metrics favouring a certain hypothesis.^10^ A holistic evaluation of a model’s performance requires a combination of performance metrics that considers different aspects of the model outputs.^11^

Across different study designs, current AI reporting checklists consistently call for clear and transparent reporting of model performance. In prediction model studies, TRIPOD-AI recommends presenting both discrimination and calibration metrics, supported by confidence intervals and, where feasible, internal and external validation to convey robustness and generalisability.^12^ For diagnostic accuracy studies, STARD-AI encourages reporting clinically interpretable measures including sensitivity, specificity, positive and negative predictive values, and summary metrics explicitly linked to the reference standard and the spectrum of cases evaluated.^13^ In trials evaluating AI systems, CONSORT-AI places importance on clear description of outcome assessment procedures and error analyses, helping to ensure that reported performance reflects clinically meaningful effects rather than artefacts of implementation or workflow integration.^14^ In line with these recommendations, the DentalCOMS guidance similarly advocates for the use of multiple complementary performance metrics, consideration of calibration where appropriate, and a specific focus on clinical relevance to support reproducibility, interpretability, and responsible translation of AI systems into dental practice.^9^

Following these initiatives regarding improving the current state of dental AI evaluation, the present study aimed to assess the knowledge and awareness of dental AI researchers and clinicians regarding performance metrics in dental image analysis.

## Methods

### Design and ethics

This was an open cross-sectional, questionnaire-based voluntary survey study of dental researchers and clinicians, administered online. Reporting adhered to the Checklist for Reporting Results of Internet E-Surveys Guidelines (CHERRIES).^15^ This research was exempt from ethics approval by the ethics committee of the Ludwig Maximilian University of Munich (Bavaria, Germany) (project number 25-0222-KB). Participants were informed about the study’s purpose, procedures, and their right to withdraw at any time. Informed consent was obtained from all participants prior to their involvement in the survey. There were no personal identifiers collected from participants to maintain the anonymity of responses.

### Participants and recruitment

The survey was open for 1 month, from 13 April to 13 May 2025. Eligible participants included researchers actively involved in dental AI image analysis, clinicians who use dental AI image analysis software, and individuals who provided informed consent to participate. Individuals not involved in dental AI research or practice, those unwilling to provide informed consent, and participants who did not complete the survey were excluded.

Initial recruitment targeted members of the ITU/WHO/WIPO Global Initiative on AI for Health Topic Group – Oral Health. This group was purposefully chosen as it comprises researchers and clinicians from multiple countries who are actively engaged in dental AI research and who possess varying levels of theoretical and practical expertise in AI for dental applications.

The survey was disseminated to this group and subsequently to wider professional networks using convenience and snowball sampling via email, LinkedIn, and relevant Discord communities, platforms commonly used for academic communication and discussion within the dental AI research community worldwide. This approach was adopted to reach a wider yet relevant population in a field where comprehensive sampling frames are limited, and expertise is distributed across interdisciplinary and international networks. As dissemination occurred across multiple online platforms and through participant sharing, the total number of individuals exposed to the survey invitation could not be determined. Consequently, view rates and participation rates could not be calculated.

No statistical weighting or other correction methods were applied to account for potential sampling bias. Given the convenience and snowball sampling strategy, findings should be interpreted in the context of a non-probability sample.

### Survey development

The questionnaire was designed to assess knowledge and awareness of AI performance metrics and was structured into distinct sections:

A) **Demographic Information:** This section collected data on participants’ age, gender, region of residence, profession/dental specialisation, years in research/clinical practice, and self-evaluation of proficiency in AI.
B) **Theoretical assessment:** This section evaluated participants’ awareness and foundational knowledge of AI performance metrics by assessing their ability to recognise commonly used metrics and identify their familiarity with specific metrics.
C) **Applied scenario-based assessment:** This section assessed participants’ applied knowledge of AI performance metrics through scenario-based questions examining their understanding of the indications, purpose, and interpretation of these metrics in dental image analysis.

The participants were also asked about their self-perceived confidence in using dental AI performance metrics after completing the knowledge questionnaire sections. Participants’ self-reported confidence was assessed for a subset of eight foundational performance metrics (Figure 2). These metrics were selected for further analysis due to their prevalence in clinical imaging literature.^9^ Other metrics detailed in Table 1, such as Matthews Correlation Coefficient (MCC) and Mean Average Precision (mAP), were evaluated for knowledge but were not included in the confidence sub-scale.

**Table 1:** Performance metrics included in the theoretical and applied knowledge-based survey questions.

Performance metric	Explanation
Accuracy	Quantifies the proportion of all predictions that the algorithm correctly classified, providing an overall measure of correctness across all outcome categories.^9^
Balanced accuracy	Accounts for class imbalance by calculating the average of sensitivity and specificity.^16^
Sensitivity/Recall	Reflects the algorithm’s ability to correctly identify cases with the target condition, indicating how effectively true positive instances are detected.^17^
Specificity	Represents the algorithm’s capacity to correctly identify cases without the target condition, capturing performance in recognising true negative instances.^9^
Precision/Positive predictive value	Describes the reliability of positive predictions by indicating the proportion of predicted positive cases that are truly positive, which is particularly important in contexts where false positives may lead to unnecessary, invasive, or costly interventions.^9^
F1 score	A statistical measure for binary classification accuracy, combining precision and recall using their harmonic mean.^17^
Area under the receiver operator characteristic curve (AUROC)	Summarises performance across decision thresholds by plotting sensitivity (the true positive rate) against the false positive rate, providing an aggregate measure of the algorithm’s ability to distinguish between classes compared with random classification.^9^
Area under the precision recall curve (AUPRC)	Summarises the trade-off between precision and recall across decision thresholds and is particularly informative for evaluating performance in datasets with low event prevalence or marked class imbalance.^18^
Intersection over Union (IoU)	Measures the spatial overlap between predicted and reference regions by dividing the area of their intersection by the area of their union, commonly used to evaluate object detection and segmentation performance in imaging tasks.^19^
Dice similarity coefficient (DSC)	Quantifies the degree of overlap between predicted and reference regions by calculating twice the intersection divided by the sum of both regions, offering a sensitive measure of segmentation accuracy in medical images.^19^
Mean average precision (mAP)	Represents the mean of average precision scores calculated across classes or detection thresholds, capturing both localisation accuracy and classification performance in object detection tasks.^20^
Matthews Correlation Coefficient (MCC)	Provides a single summary measure of classification performance that incorporates true and false positives and negatives, yielding a balanced assessment even in the presence of class imbalance.^16^

The performance metrics included in the questionnaire are depicted in Table 1.

The questionnaire was developed and validated through a multi-stage expert-led process to ensure content and face validity. Initial items were drafted collaboratively by four dental AI researchers with varying levels of experience in dental AI research, ranging from early-career to senior investigators (RR, BJ, AC, and AT). These initial items were based on the DentalCOMS framework which outlines recommended AI performance metrics in dental imaging research.^9^ These metrics were selected to reflect current reporting practice in the dental AI literature and to ensure relevance to real-world research and clinical interpretation. To establish content validity, the researchers (RR, BJ, AC, and AT) evaluated the instrument for conceptual coverage, technical accuracy, and relevance. The draft questionnaire was subsequently pilot-tested by a small expert panel comprising two experienced (SEU and FS) and two early-career (YK and JS) AI researchers. This panel assessed not only the relevance and coverage of key concepts related to AI performance metrics but also the face validity of the questionnaire to ensure clarity of language and ease of navigation on screens of variable sizes. Minor refinements were made based on their feedback.

The investigators involved in both stages of questionnaire development represented a multidisciplinary group of clinicians, clinical researchers, academic staff, and AI researchers. Collectively, the panel holds academic titles including Professor, Associate Professor, Assistant Professor, Senior Lecturer, Project coordinator, Post-doctoral researchers and a PhD Candidate with an average experience of 13 ± 5.4 years (range 8-23 years) in dental research and 5.4 ± 1.5 years (range 3-8 years) specifically in dental AI applications. This diversity in professional background and seniority ensured that the questionnaire was evaluated from both technical and clinical perspectives.

The survey was created and distributed using Qualtrics survey software (Provo, UT, USA). Data collected was anonymous as no personal identifiers (such as names, email addresses, phone numbers, etc.) were collected. The survey results were stored securely on a private cloud storage platform hosted by the University of Melbourne, Australia. The data was accessible only to the study investigators. Minimal IP address data were temporarily collected via the Qualtrics platform to identify potential duplicate submissions. Duplicate entries were identified and removed post hoc prior to analysis. Where duplicate submissions were detected, only the first complete entry was retained for analysis.

The full questionnaire is provided in Appendix. The survey comprised multiple pages, with items distributed logically by thematic section. No randomisation of items or adaptive questioning logic was implemented. Participants were able to review and modify their responses prior to final submission using the standard navigation functions within the Qualtrics platform. The anonymised data from the questionnaire was made available open source on Zenodo under the Creative Commons Attribution 4.0 International license (https://doi.org/10.5281/zenodo.18111904).^21^

Completion checks were not enforced through mandatory item responses. Participants were free to leave items unanswered. However, only fully completed questionnaires were included in the final analysis. Surveys that were terminated before completion were excluded.

### Data analysis

Completion time was not used as a criterion for data exclusion, and atypical timestamps were not assessed. This decision was made to accommodate the professional schedules of the target cohort, allowing clinicians and researchers to complete the instrument at their own pace or in multiple sessions. While timestamps were automatically recorded by the Qualtrics platform, they were not analysed as some responses spanning several hours which could reflect participants leaving the survey window open while attending to other duties rather than a lack of engagement.

The assessment comprised 12 multiple-choice questions, divided evenly between theory-based and applied scenario questions. Each question offered five possible answers. The theory-based questions allowed for multiple correct options, and participants were instructed to select all that applied. In contrast, each applied scenario question had only one correct answer.

The three possible outcomes for the theory-based questions were:

1. Correct – selected all correct options and no incorrect ones.
2. Partially correct – got at least one correct option, selected no wrong options, but didn’t select all correct answers.
3. Incorrect – selected any wrong options or missed all the correct ones.

The correct answers were scored 1 and the incorrect answers were scored 0. The partially correct outcome was only calculated for questions that had multiple correct answers. The partial score was calculated by dividing the number of correct responses selected by all possible correct responses for each question.

Data were tabulated and analysed using descriptive statistics. Continuous variables were summarised using the mean, median, standard deviation (SD), and interquartile range (IQR), and categorical variables were summarised using frequencies and percentages. Data analysis was performed using Microsoft Excel (Microsoft Corporation, Redmond, Washington, USA).

## Results

A total of 183 survey responses were received over the 1-month data-collection period. Of these, 100 responses were complete (all theory-based and applied scenario questions answered), yielding a completion rate of 54.6%. The 83 incomplete responses were excluded from analysis. As the final analytic sample comprised 100 participants, most reported percentages correspond directly to the number of participants (*n*), except where subsets are presented (eg, by specialisation or questionnaire scores). Subgroup analyses were not performed because participant numbers were unevenly distributed across subgroups, limiting the reliability and interpretability of subgroup-specific estimates.

### Demographics and perceived confidence

The majority of the participants were females (63%) and under 35 years of age (74%) (Table 2). Many of the participants were from Asia (74%) (Table 2). Across primary professions, the largest group were general dentists (46%), followed by dental specialists (39%), dental researchers (11%), oral health therapists (2%) and dental prosthetists (2%) (Table 2). Most participants were primarily based in academic institutions (74%), followed by private dental practice (20%) (Table 2).

**Table 2:** Distribution of demographic data collected during the survey (*n* = 100).

Demographic	Category	Percentage (%)
**Age (years)**	Under 25	23
	25-34	51
	35-44	15
	45-54	8
	55-64	2
	65 or above	1
**Gender**	Female	63
	Male	34
	Non-binary	1
	Transgender	2
**Region of residence**	Africa	9
	Asia	74
	Australia/New Zealand	3
	Europe	9
	North America	2
	South America	3
**Primary profession**	Dental Prosthetist/Dental Technician	2
	Dental Specialist	39
	• Conservative/Operative Dentistry	3
	• Endodontics	26
	• Oral Medicine and Pathology	1
	• Oral Radiology	2
	• Paediatric Dentistry	1
	• Periodontology	2
	• Prosthodontics	3
	• Public Health Dentistry	1
	General Dentist	46
	Oral Health Therapist	2
	Researcher in the dental field	11
**Primary workplace setting**	Academic institution	74
	Industry/Corporate	2
	Private practice	20
	Public health service	3
	Research organisation	1

**Fig. 1:**
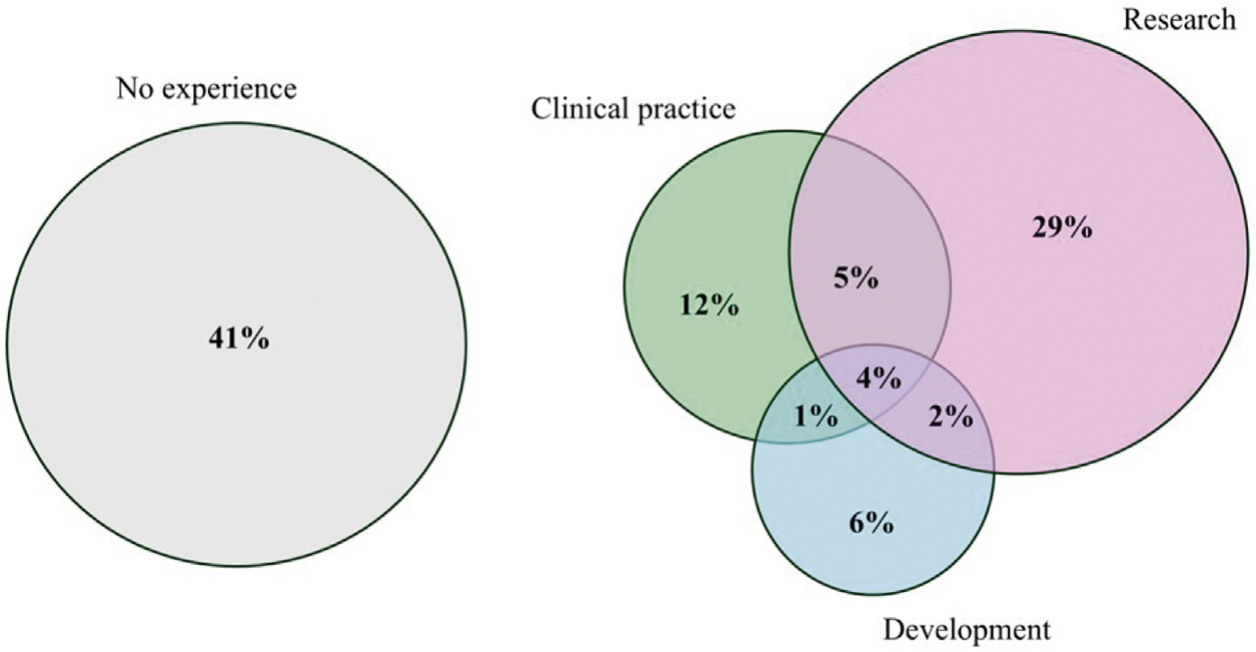
Experience in AI among participants from clinical practice research and development of AI (not to scale) (*n* = 100).

Upon being asked about their experience with AI, 41 participants (41%) reported having no experience. The remaining 59 participants (59%) indicated experience with AI in at least one of the three domains of clinical practice, research, and development. These distributions are illustrated in Figure 1 using a Venn diagram to highlight the overlaps between disciplines.

**Fig. 2:**
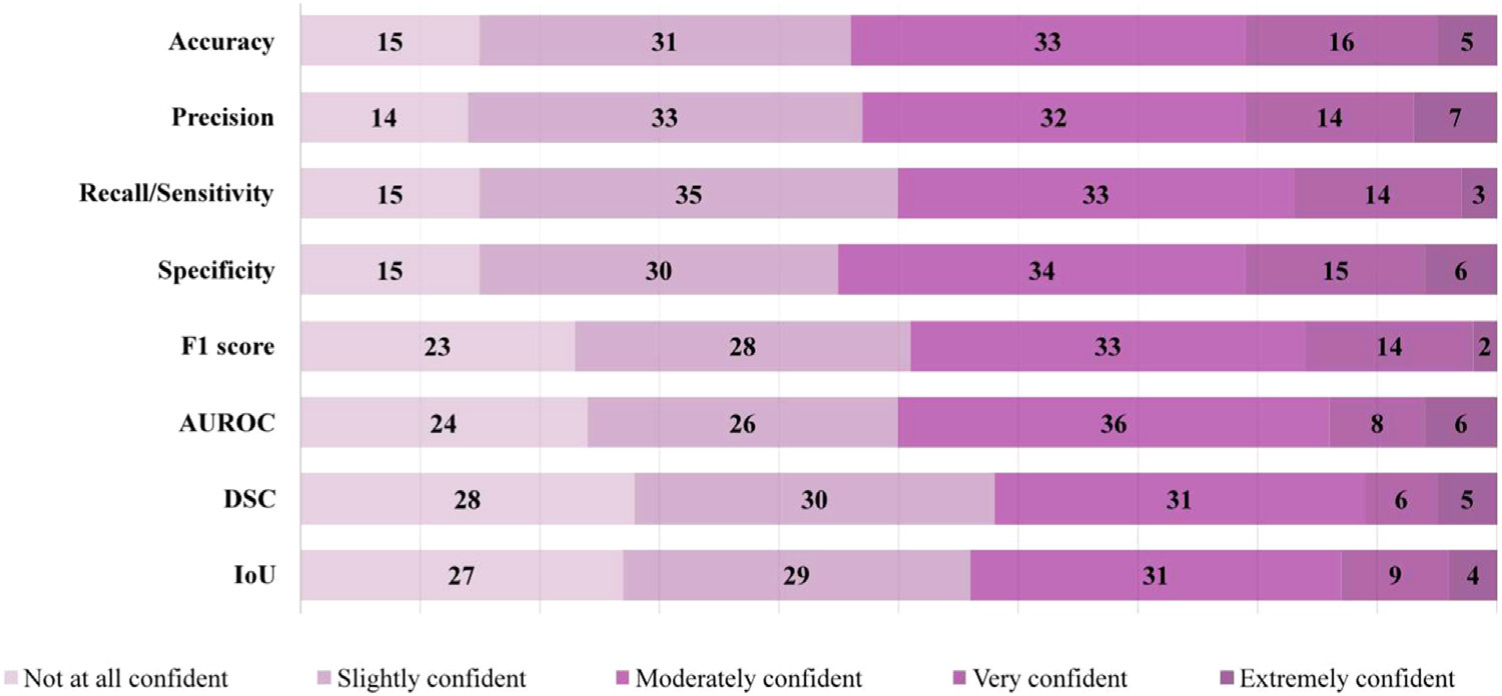
Self-reported confidence with performance metrics prior to questionnaire.

Participants’ self-reported confidence regarding common dental imaging AI performance metrics varied across measures (Figure 2). Notably, a higher number of participants reporting no confidence in metrics such as F1 score (*n* = 23, 23%), AUROC (*n* = 24, 24%), DSC (*n* = 28, 28%) and IoU (*n* = 27, 27%) compared with other metrics (Figure 2).

### Theory-based question performance

The performance for the theory-based questions (scores out of 6) had a mean score of 1.41 (23.5%), SD of 0.77 (12.8%), median of 1.3 (22.3%), and IQR of 1 (16.7%). The proportion of correct, partially correct and incorrect responses for the theory-based questions is presented in Table 3.

**Table 3:** The proportion of correct, partially correct and incorrect responses for each theory-based question (full questionnaire in Appendix 1).

Theory-based question topic	Correct answers	Correct responses (%)	Partially correct responses (%)	Incorrect responses (%)
Positive-case identification metric	Recall; Sensitivity	6%	48%	46%
Metrics for multi-threshold classification	AUPRC; AUROC	5%	50%	45%
Object detection evaluation	mAP	15%	N/A	85%
F1 score properties	Harmonic mean of precision & recall; Suitable for imbalanced datasets; Considers FP & FN	2%	54%	44%
IoU vs DSC	IoU penalises small overlaps; IoU for boxes, DSC for segmentation	1%	30%	69%
Balanced accuracy definition	Mean of sensitivity & specificity; Adjusts for class imbalance	6%	61%	34%

Across question topics the proportion of completely correct responses were consistently low, ranging from 1% to 15% (Table 3). Object detection evaluation showed the weakest performance, with only 15% of participants correctly understanding mAP and the remainder providing entirely incorrect responses (Table 3). The distinction between IoU and the DSC represented the most pronounced knowledge gap, with just 1% answering correctly and the majority demonstrating clear confusion between segmentation and detection performance metrics (69%) (Table 3). Performance was relatively stronger for balanced accuracy, where incorrect responses (34%) were less common than partial understanding (61%) (Table 3). Similarly, more than half of respondents demonstrated partial understanding (54%) of F1 score properties, although only 2 respondents got this question correct (Table 3).

### Applied question performance

The performance for the applied questions (scores out of 6) was variable with a mean score of 1.7 (28.3%), SD of 1.4 (23.0%), median of 1 (16.7%), and IQR of 1 (16.7%).

Performance was relatively strong in screening applications. Oral cancer screening achieved the highest correct response rate at 36%, where participants needed to identify recall as the appropriate metric for minimising missed cases (Table 4). Similarly, the caries detection scenario requiring precision to avoid false positives, had 37% correct responses (Table 4). These marginally better results may reflect greater familiarity with basic screening concepts in clinical practice. The orthodontic imaging task requiring DSC knowledge showed 32% correct responses, suggesting moderate understanding of spatial overlap performance metrics (Table 4). However, the more complex CBCT lesion identification scenario achieved only 22% correct responses for mean average precision, indicating substantial difficulty with multi-object detection performance metrics (Table 4).

**Table 4:** The results of survey participants (*n* = 100) for each applied question (full questionnaire in Appendix 1).

Applied question	Correct answer	Correct responses (%)	Incorrect responses (%)
Minimise missed oral cancer cases (screening)	Recall	36%	64%
Capture the full tooth extent in orthodontic radiology imaging	DSC	32%	68%
Avoid false positives in caries detection	Precision	37%	63%
Identify & localise multiple lesions in CBCT	mAP	22%	78%
Balance false positives & false negatives in cancer screening	F1 score	26%	74%
Predict rare implant failure across thresholds	AUROC	17%	83%

The most challenging applications involved the cancer screening question involving balance between false positives and negatives (F1 score) yielded 26% correct responses, while the rare implant failure prediction scenario (AUROC) produced the lowest performance at just 17% correct responses (Table 4).

### Overall performance

The overall performance which was a combination of the theory-based and applied questions had a mean score of 3.1 out of 12 (25.9%), SD of 1.8 (15.2%), median of 2.8 (22.9%), and IQR of 1.7 (14.2%). All individual scores are presented in Appendix Table 1.

Overall performance on both the theory and applied questions was generally low, with wide variation and very few participants showing strong mastery. The theory questions were particularly challenging, with many responses suggesting only partial understanding and frequent confusion between metrics that are closely related. Fully correct answers were uncommon across topics, and misunderstandings were most evident for object detection and segmentation metrics, where participants often struggled to match the metric to its intended use.

Performance on the applied questions was slightly better, but still inconsistent and often weak. Participants tended to do better when the scenarios aligned with familiar clinical screening concepts, while questions involving more complex ideas such as combined metrics, probability-based measures, or multi-object detection proved much harder to answer correctly.

### Self-perceived confidence about performance metrics

**Fig. 3:**
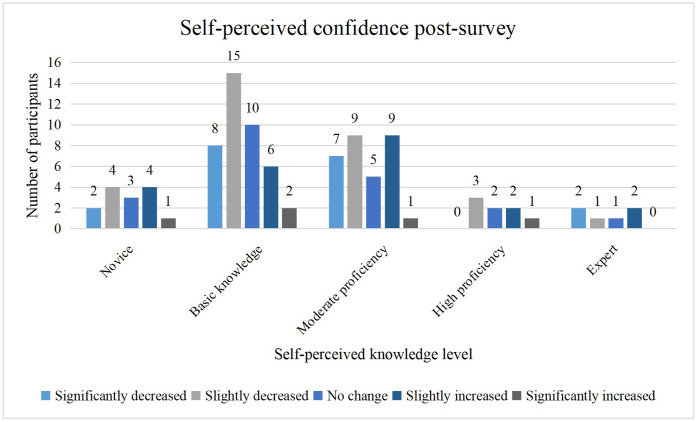
Post-survey self-perceived confidence in knowledge about dental AI performance metrics based on self-perceived proficiency.

There were varied patterns of change in self-perceived confidence across proficiency levels (Figure 3). Across all self-reported proficiency levels, most participants reported either no change or a slight shift in confidence following the survey, with extreme changes being uncommon. Increases in confidence were more frequently reported among those with novice to moderate proficiency, whereas participants at higher proficiency levels largely reported stability, with relatively few indicating substantial gains or losses (Figure 3).

## Discussion

This study assessed the knowledge and awareness of AI performance metrics among dental professionals and researchers from multiple countries. The findings revealed generally low overall performance and highlighted substantial variability in familiarity with specific performance metrics.

While participants demonstrated better recognition of widely known performance metrics such as accuracy and specificity, performance declined sharply for more complex or specialised measures, including IoU, DSC, and AUROC. These results indicated a considerable gap between awareness of basic performance metrics and the ability to recall or apply advanced evaluation measures relevant to dental AI applications. Although primary dental researchers comprised a minority of respondents (11%), at least 40% of all survey participants reported prior AI research experience, which could reflect academics who had both teaching and research appointments and may not identify as having purely research roles (Figure 1).

When viewed together, the theory-based and applied results reveal that participants often had partial theoretical knowledge of AI performance metrics. These findings suggested that misunderstandings were driven less by a complete lack of knowledge and more by confusion around terminology and closely related concepts. Knowledge-based questions showed that many respondents could recall at least one correct property or definition, but not all, pointing to gaps in depth of understanding. The applied questions, on the other hand, indicated challenges in translating this partial knowledge into correct metric selection when faced with specific clinical goals. Recent guidance by Van Calster et al^22^ has highlighted that inappropriate or poorly justified use of performance metrics remains a common weakness in clinical AI research, often obscuring true model behaviour and limiting clinical interpretability. The widespread confusion observed in this study mirrors these concerns, underscoring the need for clearer alignment between metric selection, task objectives, and transparent reporting, rather than reliance on familiar or default measures.

A possible explanation for the high error rate appears to be terminology confusion, where participants misunderstood or used similar metric terms interchangeably. For instance, the terms ‘sensitivity’ and ‘recall’ were often used synonymously, since they refer to the same measure of a model’s ability to correctly identify positive cases. However, about 48% of participants made the error of considering these as different metrics. This could lead to broader confusion if users of dental AI assume distinct meanings or fail to grasp the specific context in which these metrics are most relevant.^23^ The observed over-reliance on popular metrics such as accuracy and precision suggests a possible familiarity bias, which may contribute to poorer application of performance metrics. In scenarios where one class significantly outnumbers another (eg, rare disease diagnosis), a model that simply predicts the majority class can achieve high accuracy while being useless in practice.^24^ This highlights the necessity of employing more robust metrics like F1-score or AUPRC, which provide a more nuanced understanding of a model’s performance across different classes.^9^

Task-metric misalignment was also a critical issue, where metrics were applied to inappropriate tasks, yielding inaccurate or meaningless results. A common example is the misapplication of IoU for classification tasks. In our survey, IoU was mistakenly suggested for classification tasks in 27% of responses. IoU is a metric predominantly used in object detection and image segmentation to quantify the overlap between predicted and ground-truth spatial annotations (eg, bounding boxes or masks). Its use in a simple classification scenario, which produces categorical labels, is conceptually flawed and provides no meaningful insights into classification performance.^2^ This misalignment underscores a fundamental misunderstanding of the distinct objectives and output formats of various AI tasks.

Finally, mixing loss terms with metrics represented a conceptual confusion between training objectives and evaluation goals. Loss functions, such as cross-entropy loss, are primarily used during the model’s training phase to guide the optimization process and quantify the error between predictions and true labels.^25^ The option of cross-entropy loss was included in only one question, where 22% of the participants selected this as an answer which required a performance metric. While minimising loss is important for training a model, the loss value itself is not always an intuitive performance metric for human interpretation or for comparing different models. A model might achieve a low cross-entropy loss during training, yet still exhibit poor performance on specific, critical classes, particularly in imbalanced datasets.^25^ Performance metrics, such as accuracy, precision, or recall, were designed for the transparent and interpretable assessment of a model’s generalization capabilities on unseen data.

The findings point to a complex relationship between how participants rated their understanding of AI performance metrics and the changes they reported in their confidence. Interestingly, those with only basic knowledge formed the largest group and were also the most likely to experience a decline in confidence. This suggests that early exposure to AI metrics might make individuals more aware of the limits of their understanding. In contrast, those identifying as highly proficient (*n* = 8) or expert (*n* = 6) showed more varied responses, with neither group reporting substantial increases in confidence. This could suggest a ceiling effect or a natural stabilisation of confidence at advanced levels. Although this study was not designed to directly examine this phenomenon, the finding that participants reported relatively high confidence despite low objective knowledge scores may suggest overestimation of competence, a pattern described in the Dunning-Kruger effect.^26^ Over half of the participants (51%) reported feeling less confident about their ability to select the right performance metrics after completing the questionnaire. This drop in confidence is not necessarily negative as it may reflect a more realistic self-assessment and recognition of gaps in knowledge, which is the first step toward meaningful learning.

Participants in this study expressed interest in targeted education on task-specific metric selection to ensure that evaluation choices are better aligned with the clinical or research problem at hand. They also highlighted the need for clear metric reporting guidelines for authors, reviewers, and editors of dental AI papers, ensuring consistency and clarity in the literature. In addition, participants suggested embedding AI performance metrics into benchmarking processes, encouraging more holistic and transparent reporting rather than relying solely on a single performance number.

A key recommendation from our study is that researchers should seek information resources on the interpretation and selection of AI performance metrics.^4^ The ‘Metrics Reloaded’ framework by Maier-Hein et al^8^ was developed because commonly used metrics for validating AI algorithms often do not accurately reflect real-world biomedical image analysis problems. It provides a guide for researchers in the problem-aware selection of performance metrics for ML algorithms in biomedical image analysis. This structured approach, which captures all aspects relevant for metric selection, should also be applied to dental image analysis.^8^ Greater awareness and routine use of these guidelines would help standardise validation, reduce common misunderstandings, and strengthen the reliability of dental AI research.

The primary limitation of this study was the imbalanced sample of respondents. Consequently, the study relied solely on descriptive statistics, because analysing the underrepresented groups via subgroup or correlation analysis would increase the risk of Type II errors (false negatives) and not produce reliable results. Specifically, there were high proportions (74%) of participants from Asia, academic institutions, and under 35 years of age. This can be attributed to the convenience sampling method, initiated within the ITU/WHO/WIPO Global Initiative on AI for Health Topic Group – Oral Health and extended to other dental AI research groups and clinicians, which may have also introduced selection bias. While recruitment through these research networks facilitated reaching a highly relevant and specialised audience, it also resulted in a sample that is not fully representative of the global dental community. The participants likely had a higher baseline engagement with digital health topics, especially the younger respondents. This is possibly because this demographic may represent ‘early adopters’ who are more optimistic or knowledgeable about AI than the general dental population. Our findings should be viewed as a targeted insight into the academic sectors of the profession rather than general dentistry as a whole. Future research should prioritise targeted recruitment in private clinical practices and other underrepresented global regions to provide a more balanced perspective.

As this was a questionnaire-based survey, the data rely on self-reported proficiency and knowledge, which may be subject to response bias. The cross-sectional design provides a snapshot of knowledge and awareness at a single point in time and cannot capture changes over time or establish causal relationships. These show the importance of future studies to investigate AI performance metrics knowledge in larger cohorts over different time periods to develop a more robust understanding. Another limitation is the lack of time constraints during data collection. Participants were not required to complete the survey within a specific time limit. Thus, it was difficult to assess the attentiveness with which each participant read the questions. However, the specialised nature of the survey which required specific technical knowledge of AI performance metrics and the professional background of the participants likely encouraged a high level of deliberate engagement, reducing the risk of impulsive or random responding.

This specialised nature of the survey could have contributed to the attrition rate of 45.4% which might indicate an attrition bias. Out of the 83 incomplete survey responses, the largest drop off (34.9%, *n* = 29) occurred after collection of demographics and confidence information and just before the knowledge-based questions. A further 25.3% (*n* = 21) of dropouts occurred before engaging with any AI-related questions. Participants from academic institutions had a lower dropout rate (31.8%) compared to private practice clinicians (53.7%). This suggests the final results may be more representative of the knowledge and interest in the topic among academics rather than clinicians. However, due to the anonymous nature of the data collections a detailed analysis of incomplete responses was not possible.

A key future direction is to broaden metric choice into guidance frameworks to include evaluation of clinical utility and impact on patient outcomes. Rather than relying solely on discrimination or overlap performance metrics, studies should consider decision-analytic measures such as net benefit and decision curve analysis, which explicitly account for trade-offs between true positives and false positives in clinical contexts.^22^

## Conclusions

This study highlights substantial gaps in both knowledge and awareness of the theoretical concepts and practical applications of AI performance metrics among dental professionals and researchers. While participants demonstrated greater familiarity with basic metrics such as accuracy and specificity, performance was low when faced with more complex measures or applied clinical scenarios.

Addressing these challenges through enhanced education and a more systematic, context-aware approach to AI model evaluation is paramount for fostering the development of reliable, fair, and effective AI systems.

## Author contributions

Rishi Ramani: Conceptualization, Methodology, Formal analysis, Data Curation, Writing – Original Draft, Visualization; Bree Jones: Conceptualization, Investigation, Resources, Writing – Original Draft, Visualization; Akhilanand Chaurasia: Investigation, Resources, Writing – Review & Editing; Yeganeh Khazaei: Validation, Writing – Review & Editing; Julia Schwärzler: Validation, Writing – Review & Editing; Sergio E. Uribe: Validation, Writing – Review & Editing; Falk Schwendicke: Validation, Writing – Review & Editing; Antonin Tichy: Conceptualization, Methodology, Investigation, Resources, Writing – Review & Editing, Supervision

## Conflict of interest

The authors declare the following financial interests/personal relationships which may be considered as potential competing interests:

Given his role as a co-editor of the virtual special issue, Antonin Tichy had no involvement in the peer review of this article and had no access to information regarding its peer review. Full responsibility for the editorial process for this article was delegated to another journal editor. Other authors declare that they have no known competing financial interests or personal relationships that could have appeared to influence the work reported in this paper.

## Appendix Supplementary materials

Image, application 1

## Footnotes

Supplementary material associated with this article can be found in the online version at doi:10.1016/j.identj.2026.109566.
