# Supplementary material for: Knowledge and Awareness of Performance Metrics in Dental Image Analysis With Artificial Intelligence: A Cross-Sectional Survey of Researchers and Clinicians
Source: Int Dent J. 2026 May 6;76(4):109566. doi: 10.1016/j.identj.2026.109566 (PMC13156619; doi:10.1016/j.identj.2026.109566)
Supplement: Supplementary file 1 [file mmc1.docx]

Appendix 1

Qualtrics survey questions: Understanding AI Metrics in Dentistry: Clinician perspectives

Start of Block: Introduction

**Understanding AI Metrics in Dentistry** Hello! We would like your help understanding how dental professionals understand, interpret, and apply various metrics used to measure the performance of artificial intelligence (AI) in dentistry. Would you join us in this study by answering a quick survey? This survey is being conducted by the **ITU/WHO/WIPO Global Initiative on Artificial Intelligence for Health- Dental Diagnostics and Digital Dentistry**. It is divided into four sections: Background Information, Self-perceived Proficiency, Knowledge of Metrics and Application of Metrics. This research is**exempt from ethics approval**by **Ludwig Maximilian University of Munich Ethics Committee** (Bavaria, Germany) (project number 25-0222-KB), and will take less than **10 minutes** of your time. **Privacy:** Your answers will be confidential. We won't collect names or emails; any demographic data is safely encrypted. We use minimal IP or cookie data to prevent duplicates - this is not used for anything else and is securely deleted after analysis. **Benefits:** Your participation could help identify areas where more guidance, training, or resources may be helpful for interpreting AI metrics used in dentistry. Please answer these questions honestly, based on your current knowledge, without cross reference or checking other sources. You may opt-in to receive the final report! There are no anticipated risks. **Your Participation:** It's entirely voluntary. **Queries?** Feel free to contact us at TG-Dental@med.uni-muenchen.de  By clicking "I Agree", you confirm that you're at least 18, understand this information, and are happy to participate. Feel free to print this for your records. Thanks for considering!

- I Agree (1)

End of Block: Introduction

Start of Block: Part 1 a: Basic demographics

Part 1: Basic Demographics

Q1 Indicate your age group from the options below:

- Under 25 (1)
- 25-34 (2)
- 35-44 (3)
- 45-54 (4)
- 55-64 (5)
- 65 or above (6)

Q2 What is your gender? (Choose the option that best describes you)

- Male (1)
- Female (2)
- Non-binary (3)
- Transgender (4)
- Prefer not to say (5)

Q3 Which region best represents your location?

- Africa (1)
- Asia (2)
- Australia/New Zealand (3)
- Europe (4)
- North America (5)
- South America (6)
- Other (7) __________________________________________________

End of Block: Part 1 a: Basic demographics

Start of Block: Part 1 b: Profession and experience

Q32 Part 1: Profession and experience

Q4 Which of the following best describes your profession?

- General Dentist (1)
- Dental Specialist (2)
- Oral Health Therapist (3)
- Dental Therapist/Dental Hygienist (4)
- Dental Prosthetist/Dental Technician (5)
- Researcher in dental field (6)
- Other: (8) __________________________________________________

Display this question:

If Which of the following best describes your profession? = Dental Specialist

Q5 Please specify your clinical specialization?

- Orthodontics (1)
- Periodontology (2)
- Pediatric Dentistry (3)
- Oral Surgery (4)
- Prosthodontics (5)
- Endodontics (6)
- Oral and Maxillofacial Surgery (7)
- Oral Medicine and Pathology (8)
- Conservative/Operative Dentistry (9)
- Oral Radiology (10)
- Public Health Dentistry (11)
- Other (12) __________________________________________________

Q6 What is your primary workplace setting?

- Private practice (1)
- Academic institution (2)
- Public health service (3)
- Research organisation (4)
- Industry/Corporate (5)
- Other (6) __________________________________________________

Q7 Have you engaged with AI in any capacity, either through direct application or scholarly literature? (Select all options that apply to you)

- Yes, I have experience in AI in dental research/scientific literature. (1)
- Yes, I have experience in AI in clinical dental practice/academia. (2)
- Yes, I have experience developing AI. (3)
- I have no experience or strong familiarity with AI. (4)
- Other (5) __________________________________________________

End of Block: Part 1 b: Profession and experience

Start of Block: Part 1 c: Self evaluation of proficiency

Q34 Part 1: Self-evaluation

Q8 Overall, how would you describe your proficiency in understanding and applying AI techniques in your field?

- I am an expert (deep understanding of AI and its evaluation methods) (1)
- I am highly proficient (confident in interpreting complex metrics and applying AI methods) (2)
- I have moderate proficiency (comfortable with key concepts and common metrics) (3)
- I have basic knowledge (familiar with some concepts but not very confident) (4)
- I consider myself a novice (little to no understanding of AI) (5)

Q9 How confident are you in interpreting each metric and judging whether it is being used appropriately in research? Please rate your confidence from 1 (Not at all confident) to 5 (Extremely confident).

|  | 1 = Not at all confident (1) | 2 = Slightly confident (2) | 3 = Moderately confident (3) | 4 = Very confident (4) | 5 = Extremely confident (5) |
| --- | --- | --- | --- | --- | --- |
| **Accuracy** (1) |  |  |  |  |  |
| **Precision** (2) |  |  |  |  |  |
| **Recall** (3) |  |  |  |  |  |
| **Specificity** (4) |  |  |  |  |  |
| **F1 Score** (5) |  |  |  |  |  |
| **AUC-ROC** (6) |  |  |  |  |  |
| **Dice Similarity Coefficient** (7) |  |  |  |  |  |
| **Intersection over Union** (8) |  |  |  |  |  |

Q10 How have you gained your understanding of AI concepts and metrics? (Select all options that apply to you)

- Reading research literature and review articles (1)
- Formal education or training courses (2)
- Practical, hands-on experience with AI models (3)
- Attending workshops or conferences (4)
- Discussions with experts or colleagues (5)
- Self-directed online learning (tutorials, MOOCs) (6)
- Other (7) __________________________________________________

End of Block: Part 1 c: Self evaluation of proficiency

Start of Block: Part 2: Theoretical knowledge and awareness

Part 2: Theoretical knowledge and awareness.  In this section, you can select multiple correct answers for each question

Q11 Which metrics are used to measure the proportion of correctly identified positive cases out of all actual positive cases?

- Recall (1)
- Precision (2)
- Sensitivity (3)
- Specificity (4)
- F1 Score (5)

Q12 Which metrics are most suited to evaluate performance in classification tasks across multiple thresholds?

- Accuracy (1)
- Area Under the Precision-Recall Curve (2)
- Area Under the Receiver Operating Characteristic (ROC) Curve (3)
- Matthews Correlation Coefficient (MCC) (4)
- Mean Average Precision (5)

Q13 In object detection, which metrics evaluate both localization and classification performance?

- Intersection over Union (IoU) (1)
- Precision (2)
- Recall (3)
- Mean Average Precision (mAP) (4)
- Dice Similarity Coefficient (DSC) (5)

Q14 Which of the following statements about the F1 score are correct?

- F1 score is calculated as the harmonic mean of precision and recall. (1)
- It measures the trade-off between sensitivity and specificity. (2)
- F1 score is suitable even for imbalanced datasets where the minority class is of higher interest. (3)
- It represents the true positive rate in segmentation tasks. (4)
- Unlike specificity and precision, F1 score takes into account both false positives and false negatives. (5)

Q15 What is the difference between Intersection over Union (IoU) and Dice Similarity Coefficient (DSC)?

- DSC is more sensitive to minor segmentation errors than IoU (1)
- IoU penalizes small overlaps more than DSC. (2)
- Both metrics evaluate the boundary similarity, IoU for bounding boxes and DSC for segmentation masks. (3)
- DSC and IoU are mathematically identical metrics. (4)
- IoU is used for bounding boxes, while DSC is commonly used for pixel-based segmentation. (5)

Q16 What is balanced accuracy and why is it important in classification tasks?

- It is the arithmetic mean of sensitivity and specificity. (1)
- It is important because accuracy is sensitive to class imbalance. (2)
- It improves the precision of minority class predictions. (3)
- It adjusts for class imbalances by weighting each class equally. (4)
- It reduces overfitting in highly imbalanced datasets. (5)

End of Block: Part 2: Theoretical knowledge and awareness

Start of Block: Part 3: Applied knowledge

Q17 A model is being developed to screen clinical intra-oral in vivo microscopy for potential signs of oral cancer. The goal is to minimise missed diagnoses of oral cancer as this could lead to serious health implications. Which metric should be prioritised in evaluating this model?

- Accuracy (1)
- Precision (2)
- Recall (3)
- Intersection over Union (IoU) (4)
- Dice similarity coefficient (DSC) (5)

Q18 Orthodontics scientists at a top treatment planning company are training a model that will outline all the teeth correctly on panoramic radiographs. It is essential that the model captures the full extent of all teeth on the radiographs, including the crown and roots, to effectively guide the treatment planning. Which metric should be prioritised to evaluate this model?

- Accuracy (1)
- Mean average precision (mAP) (2)
- Dice similarity coefficient (DSC) (3)
- Area Under the Receiver Operating Characteristic (ROC) Curve (4)
- F1 score (5)

Q19 The research team at a dental AI startup is developing a new dental caries detection tool with intra-oral scanners. The goal of this model is to avoid misclassifying healthy teeth as having decay, as this could lead to unnecessary tooth preparations and treatment costs for patients. Which metric is the most important for this model that wants to avoid false positives?

- Accuracy (1)
- Precision (2)
- Recall (3)
- Sensitivity (4)
- Specificity (5)

Q20 A new deep learning model is being trialled to identify and localise several odontogenic cysts and tumours on 3D-CBCT scan data. The primary goal of this study is to correctly classify and locate each type of abnormality in the images. Which validation metric should be prioritised for this specific goal?

- Mean Average Precision (mAP) (1)
- Recall (2)
- Intersection over Union (IoU) (3)
- Accuracy (4)
- Dice Similarity Coefficient (DSC) (5)

Q21 An AI model is being developed to screen patient saliva biomarkers of oral cancer in small remote clinics with high patient workload. It is important to minimize false positives since these clinics have limited resources for follow-up tests. False negatives are also critical to avoid since undiagnosed cancer can be fatal. Which metric would provides the most balanced evaluation of model performance?

- Precision (1)
- Specificity (2)
- Accuracy (3)
- F1 Score (4)
- Dice Similarity Coefficient (DSC) (5)

Q22 A dental hospital has trained a model to predict the likelihood of patients requiring a repeat surgery following failure after dental implant placement. Since surgery in these cases are relatively rare this hospital wants a metric that will show the model’s overall ability to correctly identify high-risk patients at different thresholds to decide on resource allocation. Which is the most appropriate metric for this task?

- Matthews Correlation Coefficient (1)
- Cross entropy loss (2)
- Accuracy (3)
- F1 score (4)
- Area Under the Receiver Operating Characteristic (ROC) Curve (5)

End of Block: Part 3: Applied knowledge

Start of Block: Part 4: Next steps

Q23 Now that you have considered various AI metrics and their applications, how has your confidence in interpreting these metrics changed?

- Significantly decreased (1)
- Slightly decreased (2)
- No change (3)
- Slightly increased (4)
- Significantly increased (5)

Display this question:

If Now that you have considered various AI metrics and their applications, how has your confidence i... = Significantly decreased

Or Now that you have considered various AI metrics and their applications, how has your confidence i... = Slightly decreased

Q24 What kind of support or resources would help you feel more confident in interpreting and applying AI metrics in dentistry? (You may select more than one option)

- Online tutorials or webinars (1)
- A comprehensive guide or handbook on AI metrics for dentistry (2)
- Collaboration with experienced researchers or clinicians (3)
- Access to case studies or best-practice examples (4)
- No resources required (5)
- Other (6) __________________________________________________

End of Block: Part 4: Next steps

Start of Block: Block 7

End of Survey Thank you for completing this survey! Your time, insights, and honesty are greatly appreciated. The information you’ve provided will help us better understand the role of AI metrics in dentistry and guide future improvements in education, training, and practice.  If you have any suggestions on how we can further support professionals in understanding and applying AI metrics in dental research? Please share your thoughts.

________________________________________________________________

________________________________________________________________

________________________________________________________________

________________________________________________________________

________________________________________________________________

| 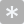 |
| --- |

If you would like to receive a summary of your responses from this survey, please list your email address below

________________________________________________________________

End of Block: Block 7
